# An updated systematic review and meta-analysis of the effects of testosterone replacement therapy on erectile function and prostate

**DOI:** 10.3389/fendo.2024.1335146

**Published:** 2024-01-26

**Authors:** Zhunan Xu, Xiangyu Chen, Hang Zhou, Congzhe Ren, Qihua Wang, Yang Pan, Li Liu, Xiaoqiang Liu

**Affiliations:** ^1^ Department of Urology, Tianjin Medical University General Hospital, Heping District, Tianjin, China; ^2^ Department of Urology, Tianjin Medical University General Hospital, Tianjin, China

**Keywords:** testosterone, late-onset hypogonadism, efficacy, safety, meta-analysis

## Abstract

**Introduction:**

Testosterone replacement therapy (TRT) is a generally accepted method treating for aging-related late-onset hypogonadism (LOH). However, the efficacy and safety of TRT remain controversial. An updated systematic review and meta-analysis aimed to determine the effectiveness and security of TRT treating for LOH.

**Methods:**

Randomized controlled trials (RCTs) of TRT for LOH were searched in the databases of Pubmed, Embase, Clinicaltrials.gov and Cochrane from 1990 to 2023 and an updated meta-analysis was conducted.

**Results:**

The results of 28 RCTs involving 3461 patients were included and scrutinized in this analysis. Among these, 11 RCTs were of long-term duration (≥12 months), while 18 RCTs were short-term studies (<12 months) comparing TRT with a placebo. TRT modalities comprised injection, oral administration, and transdermal administration. International Index of Erectile Function (IIEF) (Weighted Mean difference (WMD) 3.26; 95%; 95% confidence interval (CI) 1.65**—**4.88; P<0.0001) was obviously improved in the TRT group. International Prostate Symptom Score (IPSS) (WMD 0.00; 95% CI -0.45**—**0.45; P=1.0), Prostate Volume (PV) (WMD 0.38; 95% CI -0.64**—**1.41; P=0.46), Maximum Flow Rate (Qmax) (WMD 1.86; 95% CI -0.98**—**4.69; P=0.20), Postvoid Residual Urine Volume (PVR) (WMD 3.20; 95% CI -5.87**—**12.28; P=0.49) and Prostate-Specific Antigen (PSA) (WMD 0.08; 95% CI -0.00**—**0.17; P=0.06) were not significantly statistical between two groups.

**Conclusion:**

This meta-analysis reveals that TRT could improve the IIEF score of hypogonadal men without detriment to the IPSS score, PV, Qmax, PVR and PSA regardless of the administration method or duration of treatment.

The meta-analysis was registered at PROSPERO (CRD42023413434).

## Introduction

Late-onset hypogonadism (LOH) represents an age-related condition characterized by diminished serum testosterone levels, giving rise to a spectrum of symptoms, including hyposexuality, erectile dysfunction, muscle loss, fat gain, anemia, bone rarefaction, depression, reduced vitality, perspiration, and hot flushes (1, 2). The aging process directly correlates with LOH, potentially influencing the function or production of sex steroid hormones such as testosterone, follicular stimulating hormone, luteinizing hormon, and gonadotropinreleasing hormone (3, 4). Longitudinal studies demonstrate a gradual decline in total serum testosterone levels in men from the age of 40, 20% and 30% of men meet LOH criteria in their 60s and 70s (5, 6). LOH can significantly impact the quality of life and organ function in older men.

Testosterone is a crucial circulating androgen in males, playing a pivotal role in numerous physiological processes in different organs and systems, such as bone, muscle, fat, brain, peripheral nerves, and reproductive system (7). Testosterone replacement therapy (TRT), a generally accepted method treating for LOH, can improve the symptoms of LOH and is most usually administered by injection, oral administration and transdermal administration. Many studies have reported the benefit of TRT for LOH (8). However, some studies revealed that symptoms related to LOH may not be correlated with serum total testosterone levels (9, 10). And it is well known that androgen plays an important role in the development of benign prostatic hyperplasia (BPH) and prostate cancer (11, 12). In theory, TRT may aggravate prostate cancer and BPH. Although some meta-analyses have separately reported that TRT could promote erectile function without affecting the progression of prostate cancer and BPH, their results were dated and only a part of randomized controlled trials (RCTs) were included in their meta-analyses (8, 13, 14). The effectiveness and security of TRT for LOH remain controversial.

To clarify the available evidence, we performed an updated systematic review and meta-analysis of RCTs to determine whether TRT could promote erectile function and affect the progression of BPH and the incidence of prostate cancer.

## Methods

### Search strategy

The meta-analysis was registered at PROSPERO (CRD42023413434). RCTs which language was restricted to English from 1990 to 2023 were searched in the databases of Pubmed, Embase, Clinicaltrials.gov and Cochrane. Search terms include “androgen or testosterone”, “late-onset hypogonadism” and related expressions (**Supplementary Data Sheet 1**). Only RCTs were included in the meta-analysis after all studies were browsed by two independent reviewers. The meta-analysis was conducted according to Preferred Reporting Items for Systematic Reviews and Meta-Analyses (PRISMA) guideline (15).

### Criteria for selection

Inclusion criteria encompassed RCTs that specifically investigated the efficacy and safety of TRT for LOH. The selected RCTs were required to evaluate outcomes related to the International Index of Erectile Function (IIEF), International Prostate Symptom Score (IPSS), Postvoid Residual Urine Volume (PVR), Prostate Volume (PV), Maximum Flow Rate (Qmax) and Prostate-Specific Antigen (PSA). IIEF score was measured by IIEF-5 or IIEF.

Reviews, non-RCTs, comments, case reports, recommendations, letters, ongoing trials, protocols and studies lacking of applicable data were excluded.

### Data extraction

Two researchers extracted data independently such as first author, year, country, therapy, participants, duration, administration, method, characteristics of patients and dosage. The outcomes researched in this meta-analysis included: (1) IIEF; (2) IPSS; (3) PV; (4) Qmax; (5) PVR; (6) PSA. In instances where there were two distinct androgen administration approaches within the same RCTs, two sets of data were collected.

### Quality assessment

We used the Cochrane risk of bias tool to assess the risk of bias of the retrieved RCTs (16). The quality items were random sequence generation, allocation concealment, blinding of participants and personnel, incomplete outcome data and selective outcome reporting. The discussion among all reviewers was performed to resolve any uncertainties about the quality assessment.

### Data analysis

This meta-analysis was conducted utilizing Review Manager version 5.4. The outcomes of the included Randomized Controlled Trials (RCTs) were elucidated using Weighted Mean Difference (WMD) along with the corresponding 95% confidence interval (CI). The result of meta-analysis was statistical significant when p-value < 0.05. And we used a random effect model to calculate the differences of outcomes. The heterogeneity in data analysis was analyzed by using I^2^ statistic.

### Subgroup analysis and publication bias

Subgroup analyses was performed according to the pre-specified factors: administration, duration of follow-up and year of publication (median number). Publication bias was assessed with a funnel plot.

## Results

### Characteristics of the individual studies

**Figure 1:**
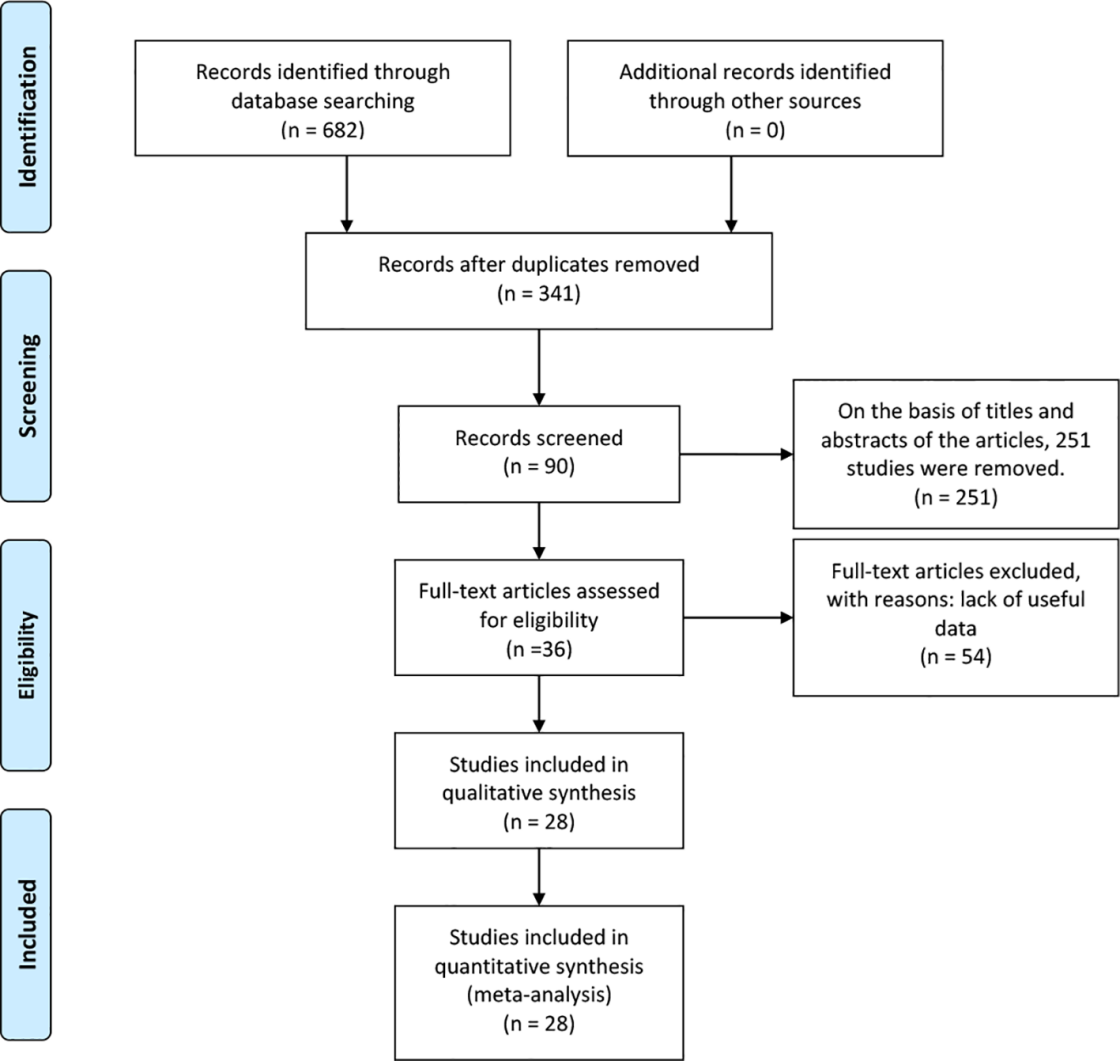
Flowchart of study inclusion.

A total of 682 articles were identified according to the searching terms from each database. 251 trails were eliminated after browsing the titles and abstracts of the articles. Fifty-four articles had a lack of valid data. Finally, 28 RCTs (17–44) that included 3253 participants (TRT group: 1710 participants and placebo group: 1543 participants) were included in our meta-analysis. Among these, 11 RCTs involved long-term treatment (≥12 months), while 18 RCTs pertained to short-term treatment (<12 months), comparing TRT with placebo (**Figure 1**). The characteristics of 28 selected RCTs are revealed in **Table 1**. The administration of TRT varied across studies, encompassing oral (3 RCTs), injection (14 RCTs), and transdermal (12 RCTs) modalities.

**Table 1:** General characteristics of the studies included.

Study	Therapy in experimental group	Therapy in control group	Sample size		Duration of ART	Administration method	Characteristics of patients	Dosage
			Experimental	Control				
**Tenover, 1992** (17)	Testosterone enanthate	Placebo	6	7	Short-term	Injection	Age ≥ 65 years, No history disease, BMI 22–29 kg/m^2^	100 mg/2 wk
**Sih et al., 1997** (18)	Testosterone cypionate	Placebo	12	10	Long-term	Injection	Age ≥ 65 years, No prostate disease	200 mg/2 wk
**Snyder et al., 1999** (19)	Testosterone patch	Placebo	45	51	Long-term	Topical	Age ≥ 65 years, No history of prostate cancer or PSA ≥ 4.0ng/mL	6 mg/d
**Kenny et al., 2001** (20)	Testosterone patch	Placebo	24	20	Long-term	Topical	Age ≥ 65 years, PSA < 4.0ng/mL	5 mg/d
**Kenny et al., 2004** (21)	Testosterone enanthate	Placebo	6	5	Short-term	Injection	Age ≥ 65 years, No history of prostate cancer or PSA ≥ 4.0ng/mL	200 mg/3 wk
**Amory et al., 2004** (22)	Testosterone enanthate	Placebo	17	18	Long-term	Injection	Age ≥ 65 years, No prostate issues	200 mg/2 wk
**Marks et al., 2006** (23)	Testosterone enanthate	Placebo	21	19	Short-term	Injection	Age 44–78 years, No prostate cancer	150 mg/2 wk
**Chiang et al., 2007** (24)	1% Testosterone gel	Placebo	20	17	Short-term	Topical	PSA < 4.0ng/mL, No prostatic masses or induration	50 mg/d
**Emmelot et al., 2008** (25)	Testosterone undecenoate	Placebo	104	103	Short-term	Oral	Age 60–80 years, No history of prostate hyperplasia and an elevated PSA	80 mg X 2/d
**Morales et al., 2009** (26)	Testosterone undecenoate	Placebo	24	28	Short-term	Oral	Age 45–70 years, No history of prostate cancer or PSA > 4.0ng/mL	80 mg X 2/d
**Chiang et al., 2009** (27)	1% Testosterone gel	Placebo	20	20	Short-term	Topical	PSA < 4.0ng/mL	50 mg/day 250 mg/4 wk
**Srinivas et al., 2010** (28)	1% Testosterone gel	Placebo	114	117	Short-term	Topical	Age ≥ 65 years, No history of prostate cancer or PSA ≥ 4.0ng/mL	50 mg/d
**Aversa et al., 2010-1** (29)	Testosterone undecenoate	Placebo	32 or 10	10	Long-term	Iniection or Oral	Age 50–65 years, T2DM and/or MetS, No history of prostate cancer	1,000 mg/12wk or 160 mg/day
**Aversa et al., 2010-2** (30)	Testosterone undecenoate	Placebo	40	10	Long-term	Injection	Age 45–65 years, No prostate or breast cancer	1000 mg/12 wk
**Giltay et al., 2010** (31)	Testosterone undecanoate	Placebo	113	71	Short-term	Injection	Age 35–70 years, No prostate or breast cancer	1,000 mg 0, 6, and every 12 wk
**Kenny et al., 2010** (32)	1% Testosterone gel	Placebo	53	46	Long-term	Topical	Age >35 years, No history of prostate cancer, PSA < 6.5ng/dL	5.0 mg/day
**Kalinchenko 2010** (33)	Testosterone undecanoate	Placebo	104	65	Short-term	Injection	Age 35–70 years, No prostate or breast cancer	1,000 mg 0, 6, 18, and 30 wk
**Amiaz et al., 2011** (4)	1% Testosterone gel	Placebo	31	32	Short-term	Topical	Age 35–70 years, Men with depression, No prostate or breast cancer	5 g/d
***Shigehara et al., 2011*** (35)	Testosterone enanthate	Blank	23	23	Long-term	Injection	PSA < 2.0ng/mL, No prostate cancer	250 mg/4 wk
**Behre et al., 2012** (36)	1% Testosterone gel	Placebo	166	155	Short-term	Topical	Age 50–80 years, PSA < 4.0ng/mL, No history of prostate or breast cancer	5 g/d
**Tan et al., 2013** (37)	Testosterone undecanoate	Placebo	56	58	Short-term	Injection	Age 40–70 years, PSA < 4 ng/mL, No prostate cancer	1,000 mg 0, 6, 18, 30, and 42 wk
**Gianatti et al., 2014** (38)	Testosterone undecanoate	Placebo	45	43	Short-term	Injection	Age 35–70 years, PSA < 4 ng/mL, No history of prostate cancer	1,000 mg 0, 6, 18, and 30 wk
**Konaka et al., 2016** (39)	Testosterone enanthate	Blank	169	165	Short-term	Injection	Age 40–90 years, No prostate cancer, PSA < 2.0 ng/mL	250 mg/4 wk
**Basaria et al., 2015** (40)	1% Testosterone gel	Placebo	129	119	Long-term	Topical	Age ≥ 60 years and IPSS ≤ 10, No prostate or breast cancer, PSA < 4 ng/mL	7.5g/day
**Paduch et al., 2015** (41)	2% Testosterone solution	Placebo	30	35	Short-term	Topical	PSA <4ng/ml, No history of prostate or breast cancer	60mg/day
**Yucel et al., 2017** (42)	Testosterone	Placebo	31	31	Short-term	Topical	PSA < 4ng/ml, No malignancy	50 mg/day
**Snyder et al., 2016** (43)	1% Testosterone gel	Placebo	234	236	Long-term	Topical	Age ≥ 65 years decreased libido, No history of prostate cancer	1,000 mg 0, 6, 18, and 30 wk
**Kato et al., 2020** (44)	Testosterone enanthate	Blank	31	29	Short-term	Injection	Age 40–90 years, No prostate cancer, PSA < 2.0 ng/mL	250 mg/4 wk

In all 28 RCTs, patients were subjected to vigilant and regular monitoring, with a protocol in place to cease treatment promptly upon the detection of signs indicative of prostate cancer or other serious complications. It is noteworthy that instances of serious complications were infrequent in the observed cohort.

### Quality of the RCTs

**Figure 2:**
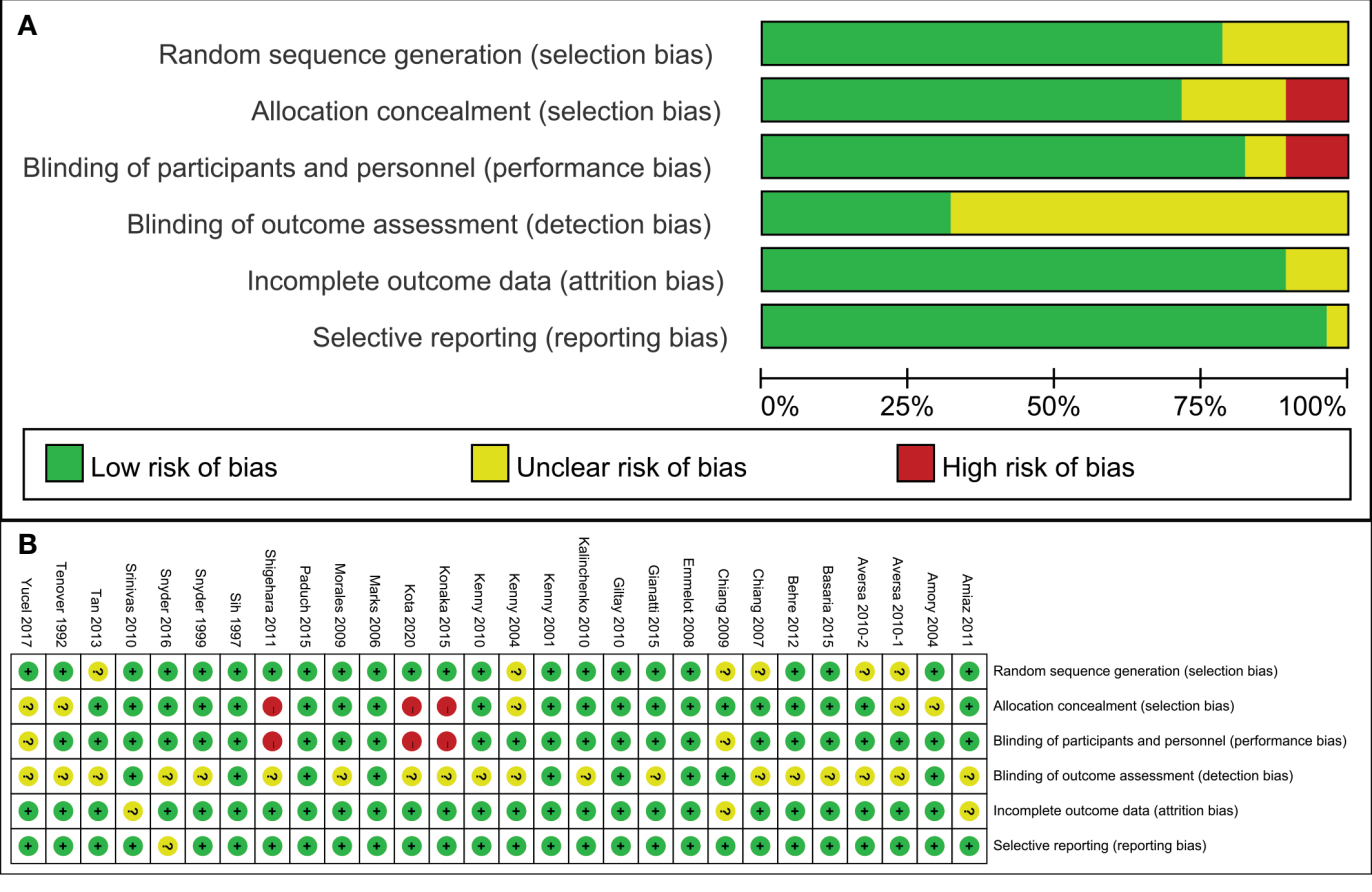
Assessment of randomized study quality. **(A)** Risk of bias graph, **(B)** Risk of bias summary.

**Figure 3:**
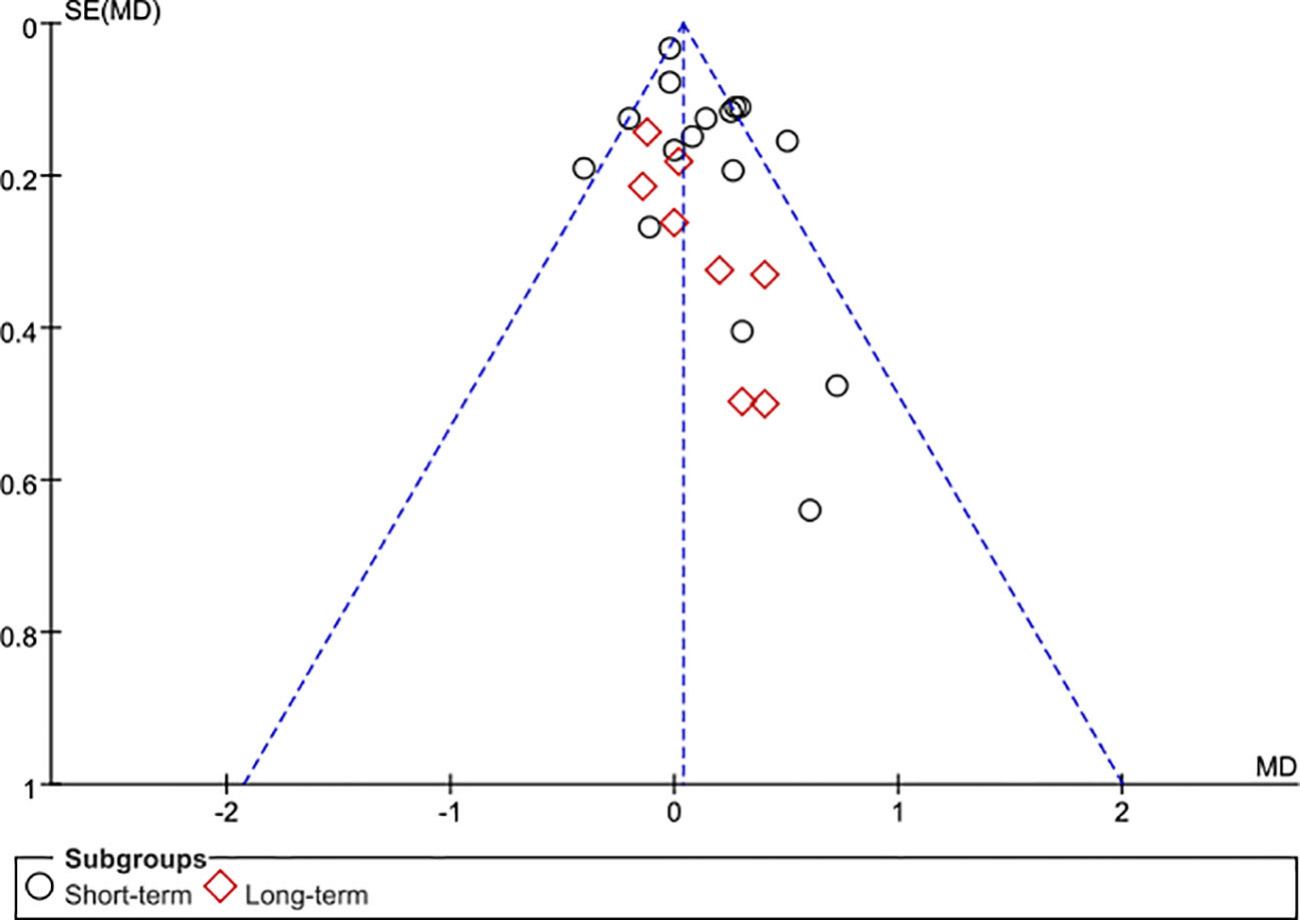
Funnel plot of the studies represented in the meta-analysis. Although 28 articles are included, 24 symbols are shown because of overlap among the articles in reporting TRT. MD, mean difference; SE, standard error.

The 28 articles included in the study were RCTs. The assessment of risk of bias of studies was showed in **Figure 2**. Three studies showed selection bias and performance bias. A funnel plot was constructed to assess publication bias in the meta-analysis of PSA with a stent indwelling, including all 24 studies. The plot indicated the an absence of publication bias (**Figure 3**).

## Result of meta-analysis

### IIEF

**Figure 4:**
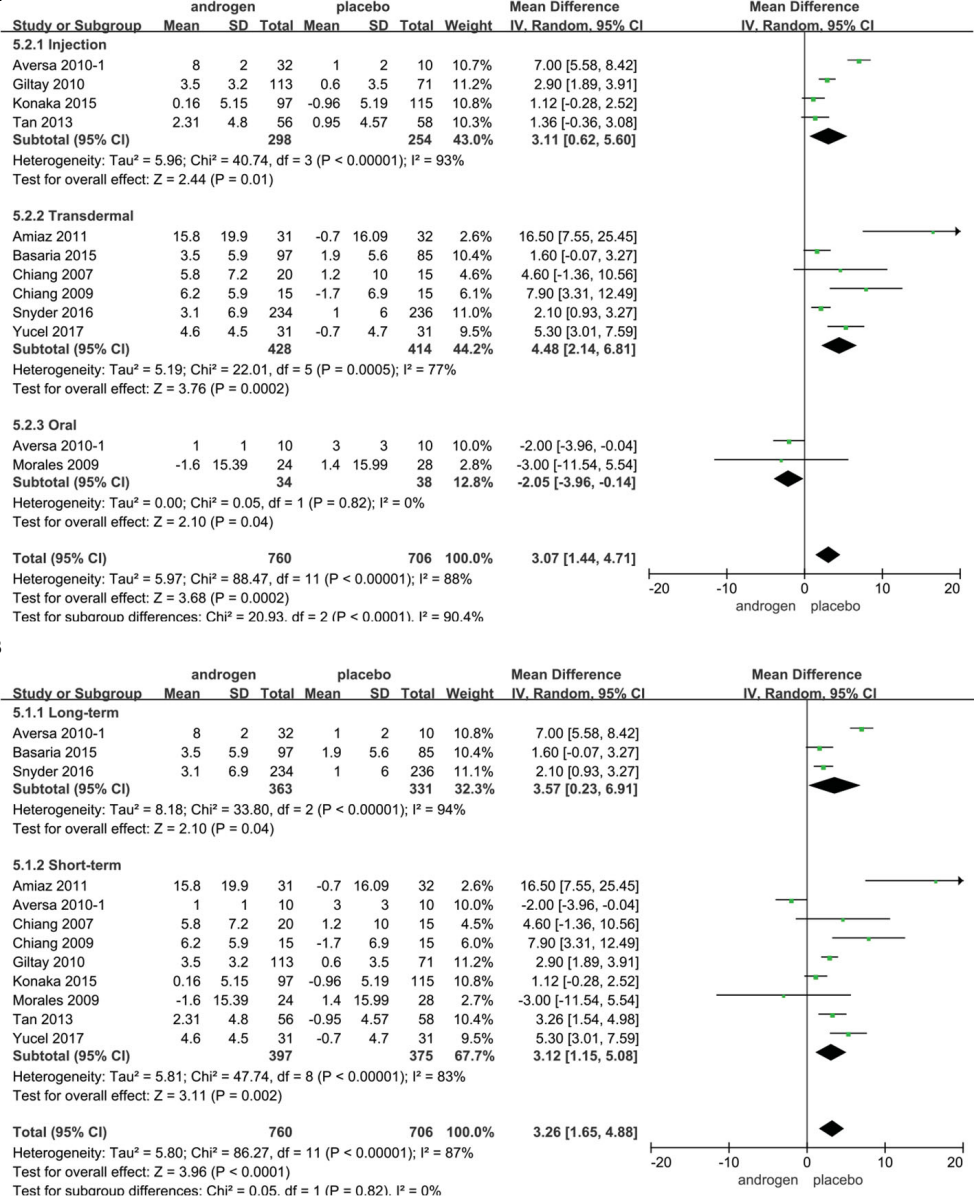
Forest plots of IIEF. **(A)** Subgroup analysis of administration of TRT, **(B)** Subgroup analysis of duration of TRT.

Twelve RCTs full of 1466 patients were applied to assess the IIEF score (TRT group: 760 participants and placebo group: 706 participants). We adopted a random-effects model to evaluate the difference in IIEF between two groups (WMD 3.26; 95% CI 1.65**—**4.88; P<0.0001). The forest plots vividly illustrated a notable improvement in the IIEF score within the TRT group compared to the placebo group (**Figure 4**).

According to the route of administration, 12 studies were divided into three groups for subgroup analyses: five studies employed injection, transdermal application was utilized in six studies, two studies involved oral treatment. The results indicated a significant increase in the IIEF score with TRT compared to placebo regardless of administration method (**Figure 4A**).

Subgroup analyses were conducted after dividing the 12 studies into two groups based on the duration of treatment: 3 RCTs constituted the long-term treatment group (≥12 months), while 9 RCTs formed the short-term treatment group (<12 months). TRT was more likely to increase the IIEF score than placebo. And the results were not associated with the duration of treatment (**Figure 4B**).

Additionally, considering the year of publication, the 12 studies were stratified into two groups for subgroup analyses: 3 RCTs were published before 2010, and 9 RCTs were published after 2010. Notably, the subgroup analysis exclusively for RCTs published after 2010 demonstrated a significant enhancement in erectile function with TRT (**Supplementary Figure 1**).

### IPSS

**Figure 5:**
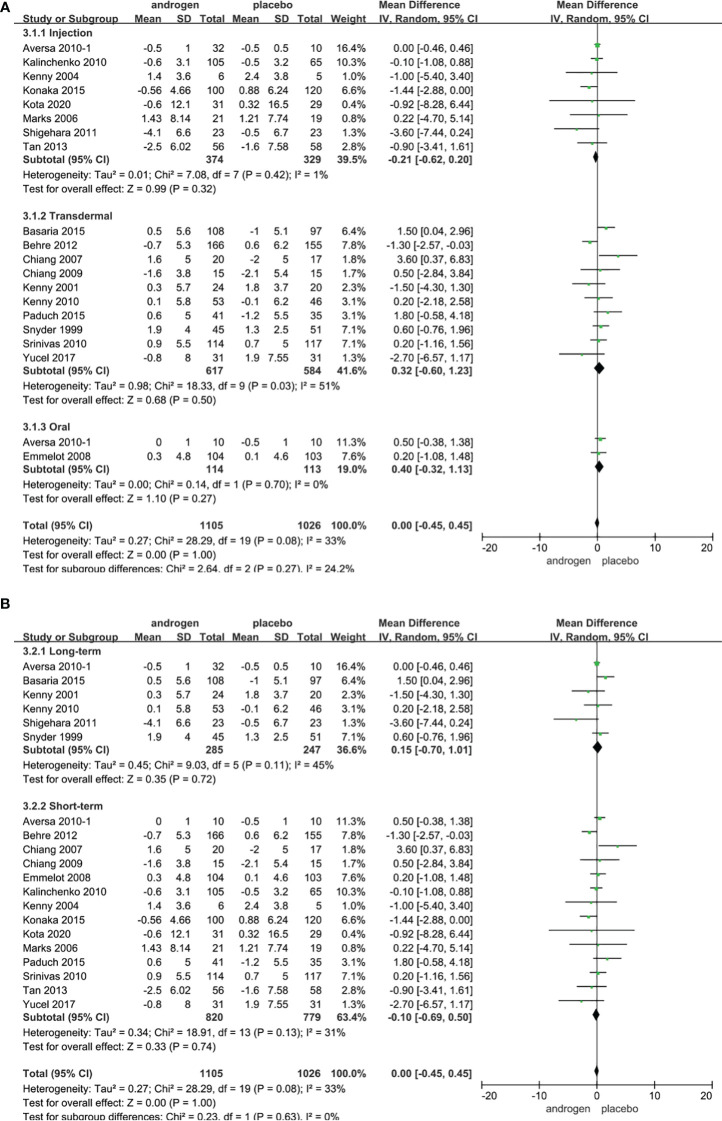
Forest plots of IPSS. **(A)** Subgroup analysis of administration of TRT, **(B)** Subgroup analysis of duration of TRT.

Twenty RCTs, involving 2131 patients (TRT group: 1104 participants; placebo group: 1026 participants), were utilized to assess the IPSS. Employing a random-effects model, we assessed the difference in IPSS between two groups (WMD 0.00; 95% CI -0.45**—**0.45; P=1.0). The results revealed that there was no significant difference in the change of the IPSS between the two groups (**Figure 5**).

According to the route of administration, the 20 studies were categorized into three groups for subgroup analyses: 8 studies were administered by injection, transdermal application was utilized in 10 studies, and 2 studies involved oral treatment. The results indicated that the changes in IPSS were comparable between two groups regardless of route of administration (**Figure 5A**).

Subgroup analyses were conducted after dividing 20 studies into two groups according to the duration of treatment in our studies: 6 RCTs constituted the long-term treatment group (≥12 months) and 14 RCTs formed the short-term treatment group (<12 months). There was no statistical difference in the IPSS between the two groups. And the result was not associated with the duration of treatment (**Figure 5B**).

Considering the year of publication, the 20 studies were stratified into two groups for subgroup analyses: 7 RCTs were published before 2010, 13 RCTs were published after 2010. The results showed that TRT could not significantly alter IPSS score, irrespective of the year of publication (**Supplementary Figure 2**).

### PV

**Figure 6:**
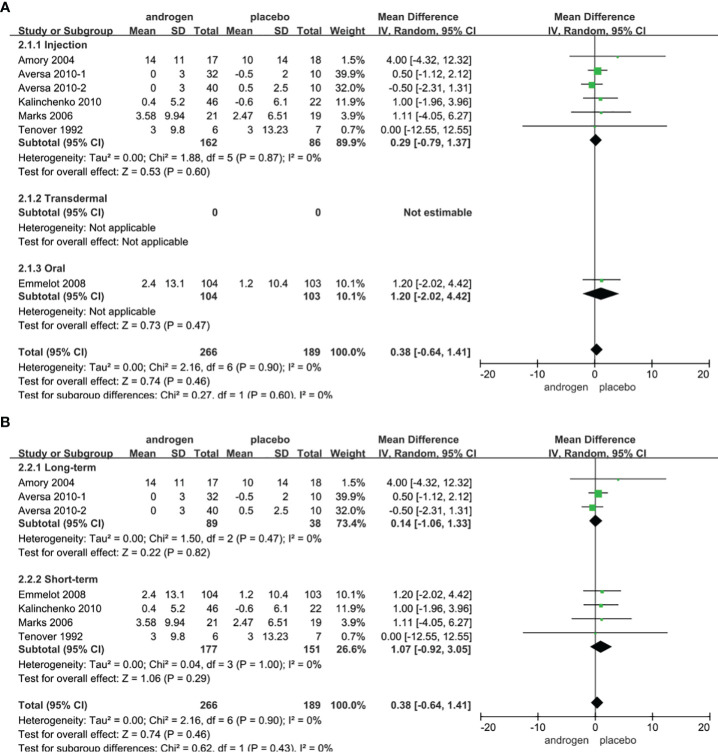
Forest plots of PV. **(A)** Subgroup analysis of administration of TRT, **(B)** Subgroup analysis of duration of TRT.

Seven RCTs full of 455 patients were applied to assess the score of PV (TRT group: 266 participants and placebo group: 189 participants). We adopted a random-effects model to evaluate the difference in the change of PV between two groups (WMD 0.38; 95% CI -0.64**—**1.41; P=0.46). The forest plots revealed that there was no significant difference in the changes of PV in both groups (**Figure 6**).

According to the route of administration in our studies, 7 studies were divided into three groups for subgroup analyses: 6 studies employed administered by injection, transdermal application was used in 0 studies, 1 studies involved oral treatment. The results showed that the changes of PV were similar after treatment in both groups regardless of mode of administration (**Figure 6A**).

Subgroup analyses were conducted after dividing the seven studies into two groups based on the duration of treatment: 3 RCTs constituted the long-term treatment group (≥12 months) and 4 RCTs formed short-term treatment group (<12 months). There was no apparent difference in the changes of PV between the two groups. And the results were not associated with the duration of treatment (**Figure 6B**).

Considering the year of publication, the 7 studies were divided into two groups for subgroup analyses: 4 RCTs were published before 2010, 3 RCTs were published after 2010. The results showed that the change of PV was similar between two groups regardless of year of publication (**Supplementary Figure 3**).

### Qmax

**Figure 7:**
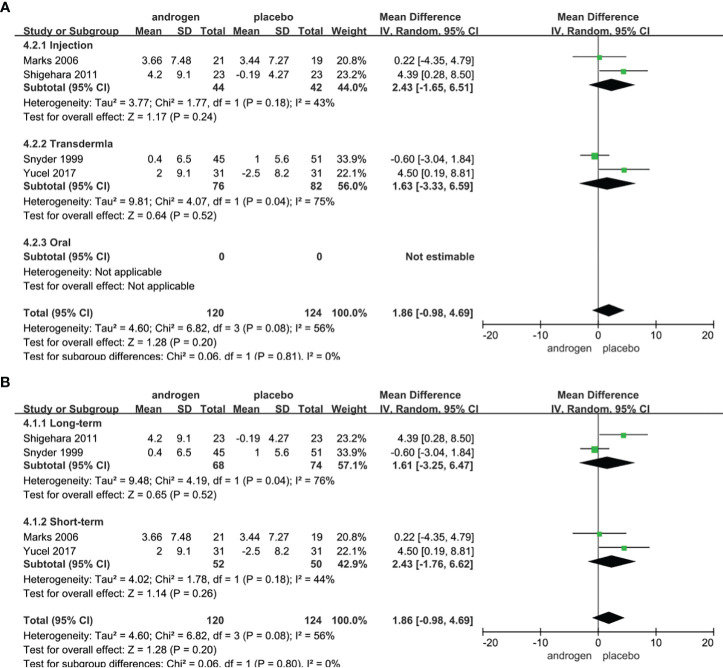
Forest plots of Qmax. **(A)** Subgroup analysis of administration of TRT, **(B)** Subgroup analysis of duration of TRT.

Four RCTs full of 244 patients were used to evaluate the score of Qmax (TRT group: 120 participants and placebo group: 124 participants). We adopted a random-effects model to assess the difference of PSA between two groups (WMD 1.86; 95% CI -0.98**—**4.69; P=0.20). The meta-analyses revealed that there was no significant difference in the changes of Qmax between the two groups (**Figure 7**).

According to the route of administration in our studies, 4 studies were divided into three groups for subgroup analyses: 2 studies were administered by injection, transdermal application was used in 2 studies, no studies received oral treatment. The results declared that the changes of Qmax were similar after treatment in both groups regardless of mode of administration (**Figure 7A**).

Subgroup analyses were conducted after dividing the four studies into two groups based on the duration of treatment: 2 RCTs constituted the long-term treatment group (≥12 months) and 2 RCTs formed the short-term treatment group (<12 months). The subgroup analyses showed there was a similar change in the Qmax between the two groups (**Figure 7B**).

Considering the year of publication, the 4 studies were divided into two groups for subgroup analyses: 2 RCTs were published before 2010, 2 RCTs were published after 2010. The result of subgroup analysis of RCTs published after 2010 showed an improvement in Qmax in TRT group (**Supplementary Figure 4**).

### PVR

**Figure 8:**
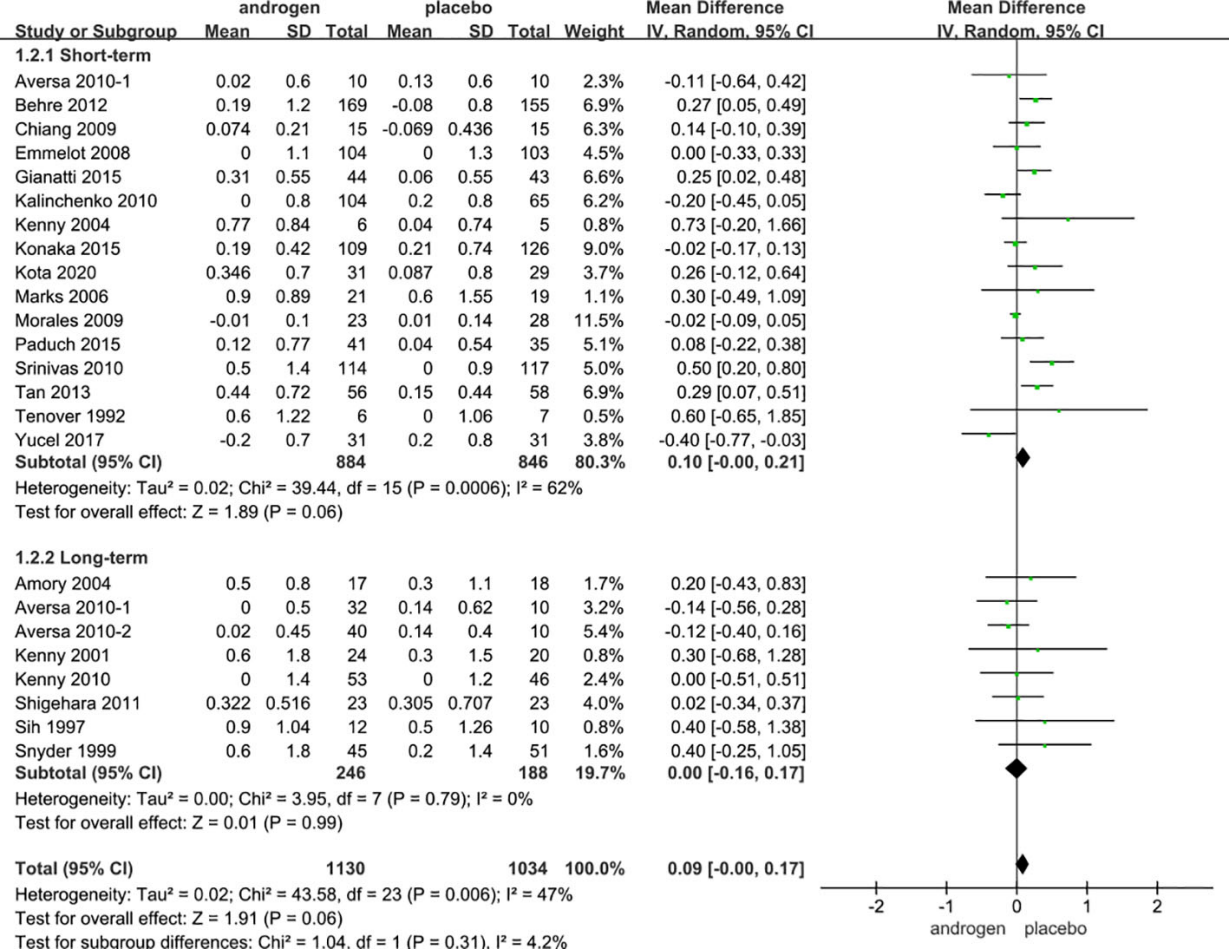
Forest plots of PVR: Subgroup analysis of duration of TRT.

Three RCTs full of 158 patients were applied to assess the score of PV (TRT group: 74 participants and placebo group: 84 participants). We adopted a random-effects model to evaluate the difference of PVR between two groups (WMD 3.20; 95% CI -5.87**—**12.28; P=0.49). The meta-analyses revealed that the improvements in the PVR were similar between the two groups (**Figure 8**).

Subgroup analyses were conducted after dividing the three studies into two groups based on the duration of treatment: 2 RCTs constituted the long-term treatment group (≥12 months), and 1 RCT formed the short-term treatment group (<12 months). The subgroup analyses showed the improvements in the PVR were not statistically significant (**Figure 8**).

Considering the year of publication, 3 studies were divided into two groups for subgroup analyses: 2 RCTs were published before 2010, and 1 RCTs were published after 2010. The results declared that no significant changes in PVR in either group (**Supplementary Figure 5**).

### PSA

**Figure 9:**
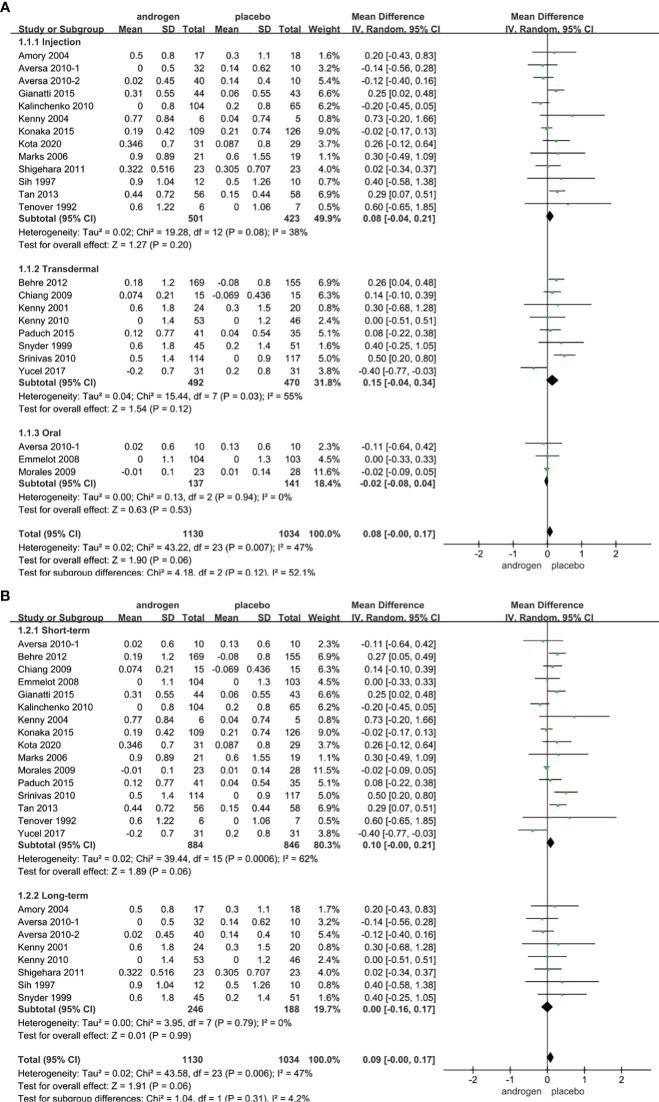
Forest plots of PSA. **(A)** Subgroup analysis of administration of TRT, **(B)** Subgroup analysis of duration of TRT.

Twenty four RCTs full of 2164 patients were applied to assess the score of PSA (TRT group: 1130 participants and placebo group: 1034 participants). We adopted a random-effects model to assess the difference of PSA (WMD 0.08; 95% CI -0.00**—**0.17; P=0.06). The forest plots revealed that there was no significant difference in the changes of the PSA in both groups (**Figure 9**).

According to the route of administration in our studies, 24 studies were divided into three groups for subgroup analyses: 13 studies employed injection, transdermal application was used in 8 studies, 3 studies involved oral treatment. The results stated that the changes in PSA were similar after treatment in both groups regardless of mode of administration (**Figure 9A**).

Subgroup analyses were conducted after dividing the twenty-four studies into two groups based on the duration of treatment: 8 RCTs constituted the long-term treatment group (≥12 months), and 16 RCTs formed the short-term treatment group (<12 months). The subgroup analyses showed the influences on PSA were not statistically significant. And the result was not associated with the duration of treatment (**Figure 9B**).

Considering the year of publication, 3 studies were divided into two groups for subgroup analyses: 10 RCTs were published before 2010, 14 RCTs were published after 2010. The result declared that the differences in PSA levels were similar in two groups regardless of year of publication (**Supplementary Figure 6**).

## Discussion

The systematic review and meta-analysis included 28 RCTs (3253 patients) published since 1990. These RCTs investigated the impacts of TRT on erectile function, prostate growth, and prostate cancer in individuals with LOH and were performed in different countries worldwide. However, there existed variability in the inclusion criterion for testosterone levels among the RCTs due to a lack of consensus in defining LOH across different medical associations. The European Urological Association recommends a total testosterone level below 300 ng/dL as the diagnosis of hypogonadism (45), the American Urological Association considers 12 nmol/L total testosterone (3.5 ng/mL) represents a reliable threshold to diagnose LOH (46), and the Endocrine Society recommends the reasonable cut-off of total testosterone in men as 264 ng/dL (9.2 nmol/L) (47). This variation in diagnostic criteria underscores the lack of uniformity in defining LOH across different medical entities, potentially contributing to the heterogeneity observed in the included RCTs.

In our analysis, TRT could statistically increase IIEF score than placebo, irrespective of the duration of treatment or the administration method. Hisanori et al. (8) also demonstrated that TRT could benefit erectile function in patients with LOH, especially those receiving long-term TRT with testosterone gel (>30 weeks) and without comorbidities. This improvement was noteworthy, especially since intramuscular and oral doses were each utilized in only one study. A systematic review including 6 RCTs reported that the improvements of sexuality and erectile function were significant after treatment of TRT compared with placebo (48). Corona et al. (49) declared that there was an obvious increase in the IIEF score for the TRT group compared with the placebo group; in addition, the change in IIEF-EF score was more significant in patients with total testosterone <8 nmol/L than those with total testosterone <12 nmol/L (49). A prior meta-analysis involving 17 RCTs in men with LOH demonstrated that TRT was more likely to improve various aspects of sexual function, including morning erection, erectile function, sexual intercourse, sexual motivation, sexual satisfaction, sexual thoughts, and total erection (50). However, the subgroup analyses based on the year of publication showed that TRT could significantly promote erectile function only after 2010, possibly due to the limited number of articles published before that year. The most common way to assess erectile function in patients with hypogonadism was the questionnaire, and the results are subjective. However, there was no research diagnosed ED and assessed the efficacy of TRT by an objective evaluation method such as a nocturnal penile tumescence and rigidity analysis. Additionally, it should be emphasized that some participants in the study did not suffer from ED.

Our analysis of 23 RCTs involving 2328 patients revealed that the changes in IPSS, PV, Qmax and PVR were not statistically significant between the two groups. Only the subgroup analysis of RCTs published after 2010 showed an improvement in Qmax in TRT group. However, it is essential to note that further research is warranted due to the limited number of RCTs available for this specific analysis. Shigehara et al. (51) observed that TRT could significantly benefit IPSS and Qmax for LOH patients with mild BPH. Pearl et al. (52) performed a retrospective study including 120 patients and reported that IPSS decreased by an average of 7.42 for patients with severe IPSS after treatment of TRT. However, it remains unclear whether the patients were taking medications to treat LUTS during the study period. Kohn et al. (13) reported that there was no statistical difference in IPSS in the TRT group compared with the placebo group. Saad et al. (53) revealed that TRT could influence the PV during the first 3 months of treatment but had little effect on PV after 12 months. They also reported that the changes in PV after 12 months were likely related to aging rather than testosterone therapy. A meta-analysis performed by Cui (54) suggested that TRT showed no significant changes in IPSS, PV and Qmax compared with placebo. Other several studies also showed non-statistically significant worsening of Qmax and PVR caused by BPH during TRT (55, 56). Our meta-analysis assessed the safety of both long-term and short-term TRT for prostate growth. Importantly, all 23 RCTs included patients both with and without prostate enlargement at baseline. The conclusive findings indicate that TRT does not promote prostate growth in patients with hypogonadism.

Our study also elucidated that there was no statistically significant change in PSA between the two groups. Early doctors considered that the use of testosterone could develop the growth of prostate cancer and testosterone was forbidden for patients with possible prostate cancer (57). However, in clinical practice, the PSA level does not necessarily increase with rising testosterone levels when elevated to eugonadal levels (58). Several studies revealed that TRT could initially increase the serum PSA level especially in patients with extremely low testosterone levels before treatment, and this effect to be weakened with time (59, 60). Morgentaler proposed a “prostate saturation model” to explain these findings, he considered that TRT influences PSA and prostate growth only when the levels of testosterone are extremely low, once testosterone levels are increased to a specific threshold concentration which was much lower than the physiologic testosterone levels observed in the clinical work, TRT would not influence PSA and prostate growth (60). Santella showed that TRT would not increase the risk of prostate cancer in men with LOH (61). Kato observed that TRT increased the PSA level (44). However, the elevation of PSA levels was within the normal range and the time of administration of testosterone was only 6 months. Konaka revealed that the difference in changes of PSA levels between two groups was not obvious with an increase in the duration of testosterone administration to 52 weeks (39). All 29 RCTs in this meta-analysis rigorously and regularly monitored patients. Treatment was stopped when there are signs of suspected prostate cancer or other serious complications. The PSA levels were normal for all patients at baseline, and no patient underwent a prostate biopsy at the start of the study. Patients with prostate cancer or a history of prostate and breast cancer were excluded. At the end of the study, there was no significant difference in the positive rate of prostate biopsy between the two groups. However, rigorous monitoring is still indispensable in the course of treatment.

In addition to concerns about the impact on the prostate growth and prostate cancer, side effects of TRT may include cardiovascular risk, erythrocytosis, increased hemoglobin and a decrease in high-density lipoprotein (HDL) cholesterol. Basaria’s study showed that TRT would significantly increase cardiovascular risk compared to the placebo group (62). Similarly, Vigen revealed that patients treated with TRT after coronary angiography suffered significant cardiovascular events compared with patients treated without TRT (63). However, a meta-analysis reported that TRT had no effect on the cardiovascular events (64). Another meta-analysis also demonstrated that TRT has no significant effect on cardiovascular events, but TRT would result in an increase in hemoglobin and hematocrit and a decrease in HDL cholesterol (65). Therefore, it is imperative to consider periodic monitoring of these indicators in patients undergoing TRT.

In clinical practice, many urologists express concerns that TRT might affect the progression of BPH and the incidence of prostate cancer. Our meta-analysis, however, contradicts these concerns, demonstrating that TRT not only enhances erectile function but also does not exacerbate BPH or increase the incidence of prostate cancer. This results could provide more evidence for the clinical utilization of TRT for treating LOH and guiding the dietary recommendations for LOH patients. Lu’s report also reported that the intake of plant-based diet could be beneficial to erectile function in men over 45 yeas old and without impacting total testosterone level (66, 67). Therefore, incorporating a diet that fosters testosterone production and includes plant-based elements could be beneficial for LOH patients.

In our meta-analysis, exclusive focus was placed on RCTs, each of which exhibited high methodological quality. Unlike previous meta-analyses that individually addressed the effects on LUTS, PSA, or erectile function, our study encompassed a substantially larger cohort of patients and RCTs. Notably, we unveiled comprehensive insights into the alterations in LUTS, PSA, erectile function, and additionally, PVR – an aspect often overlooked in prior meta-analyses. Our subgroup analyses further corroborated the consistency of our findings, reinforcing the robustness and reliability of our results. However, it is essential to acknowledge certain limitations in our study. Variability in inclusion criteria was noted across studies; while some studies exclusively considered patients with low serum testosterone levels, others did not account for hypogonadal symptoms. Various factors such as testosterone levels, age, underlying diseases, prostatitis, and bladder dysfunction can influence LUTS, PSA, or erectile function. The number of RCTs reporting PVR or Maximum Flow Rate (Qmax) included in the analysis, along with the sample sizes of these studies, were relatively small, potentially limiting the generalizability of our findings. Additionally, differences in the measurements of testosterone levels contributed to potential heterogeneity in the results.

In this meta-analysis of RCTs, TRT could improve the erectile function of hypogonadal men. But there are no significant difference in changes of IPSS, PSA, PV, Qmax and PVR regardless of the administration method or duration of treatment among men in TRT group versus those in placebo group.

## Data availability statement

The original contributions presented in the study are included in the article/**Supplementary Material**. Further inquiries can be directed to the corresponding authors.

## Author contributions

ZX: Methodology, Writing – original draft. XC: Methodology, Writing – original draft. HZ: Formal analysis, Writing – original draft. CR: Formal analysis, Writing – original draft. QW: Data curation, Writing – original draft. YP: Data curation, Writing – original draft. LL: Conceptualization, Writing – review & editing. X-QL: Conceptualization, Funding acquisition, Writing – review & editing.

## Supplementary material

Supplementary Figure 1 Subgroup analysis of IIEF based on the year of publication.

Supplementary Figure 2 Subgroup analysis of IPSS based on the year of publication.

Supplementary Figure 3 Subgroup analysis of PV based on the year of publication.

Supplementary Figure 4 Subgroup analysis of Qmax based on the year of publication.

Supplementary Figure 5 Subgroup analysis of PVR based on the year of publication.

Supplementary Figure 6 Subgroup analysis of PSA based on the year of publication.
