# Supplementary material for: An updated systematic review and meta-analysis of the effects of testosterone replacement therapy on erectile function and prostate
Source: Front Endocrinol (Lausanne). 2024 Jan 26;15:1335146. doi: 10.3389/fendo.2024.1335146 (PMC10853420; doi:10.3389/fendo.2024.1335146)
Supplement: Supplementary file 1 [file DataSheet_1.docx]

**Supplementary Data Sheet 1: Search strategies**

**Pubmed**

#1 (((((((((testosterone[Mesh]) OR (testosterone replacement therapy[Title/Abstract])) OR (testosterone therapy[Title/Abstract]))) OR (testosterone administration[Title/Abstract])))) OR (testosterone treatment[Title/Abstract]))))) OR (testosterone supplementation[Title/Abstract])))))) OR (androgen[Title/Abstract]))))))) OR (androgen replacement therapy[Title/Abstract])))))))) OR (androgen administration[Title/Abstract]))))))))) OR (androgen therapy[Title/Abstract])

#2 (((((late-onset hypogonadism[Mesh]) OR (LOH[Title/Abstract])) OR (hypogonadism[Title/Abstract]))) OR (androgen deficiency[Title/Abstract])))) OR (hypogonadal men[Title/Abstract]))))) OR (testosterone deficiency[Title/Abstract])

#3 #1 AND #2

#4 (randomized[Title/Abstract])

#5 #3 AND #4

**Embase**

#1 'testosterone'/exp OR 'testosterone replacement therapy':ab,ti OR 'testosterone therapy':ab,ti OR 'testosterone administration':ab,ti OR 'testosterone treatment':ab,ti OR 'testosterone supplementation':ab,ti OR 'androgen':ab,ti OR 'androgen replacement therapy':ab,ti OR 'androgen administration':ab,ti OR 'androgen therapy':ab,ti

#2 'late-onset hypogonadism'/exp OR 'LOH':ab,ti OR 'hypogonadism':ab,ti OR 'androgen deficiency':ab,ti OR 'hypogonadal men':ab,ti OR 'testosterone deficiency':ab,ti

#3 #1 AND #2

#4 'randomized':ab,ti

#5 #3 AND #4

**Clinicaltrials.gov**

"androgen" OR "testosterone replacement therapy" OR "testosterone therapy" OR "testosterone administration" OR "testosterone treatment" OR "testosterone supplementation" OR "androgen" OR "androgen replacement therapy" OR "androgen administration" OR "androgen therapy" AND "late-onset hypogonadism" OR "LOH" OR "hypogonadism" OR "androgen deficiency" OR "hypogonadal men" OR "testosterone deficiency" AND "randomized"

**Cochrane**

"androgen" OR "testosterone replacement therapy" OR "testosterone therapy" OR "testosterone administration" OR "testosterone treatment" OR "testosterone supplementation" OR "androgen" OR "androgen replacement therapy" OR "androgen administration" OR "androgen therapy":ti,ab,kw AND "late-onset hypogonadism" OR "LOH" OR "hypogonadism" OR "androgen deficiency" OR "hypogonadal men" OR "testosterone deficiency":ti,ab,kw AND "randomized":ti,ab,kw
