# Supplementary figures and images for: An updated systematic review and meta-analysis of the effects of testosterone replacement therapy on erectile function and prostate
Source: Front Endocrinol (Lausanne). 2024 Jan 26;15:1335146. doi: 10.3389/fendo.2024.1335146 (PMC10853420; doi:10.3389/fendo.2024.1335146)

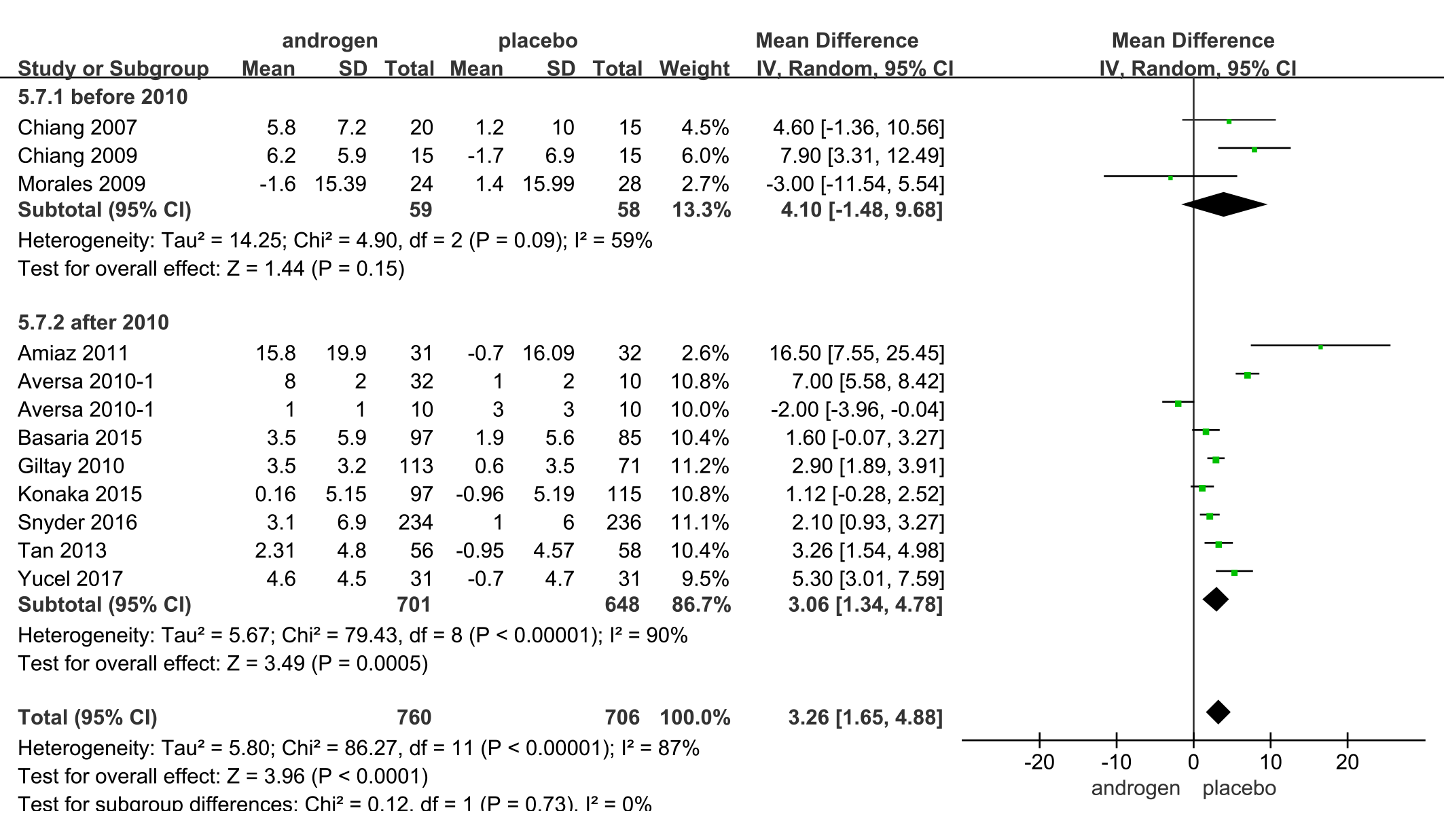

Supplement: Supplementary Figure 1 — Subgroup analysis of IIEF based on the year of publication. [file Image_1.tif]

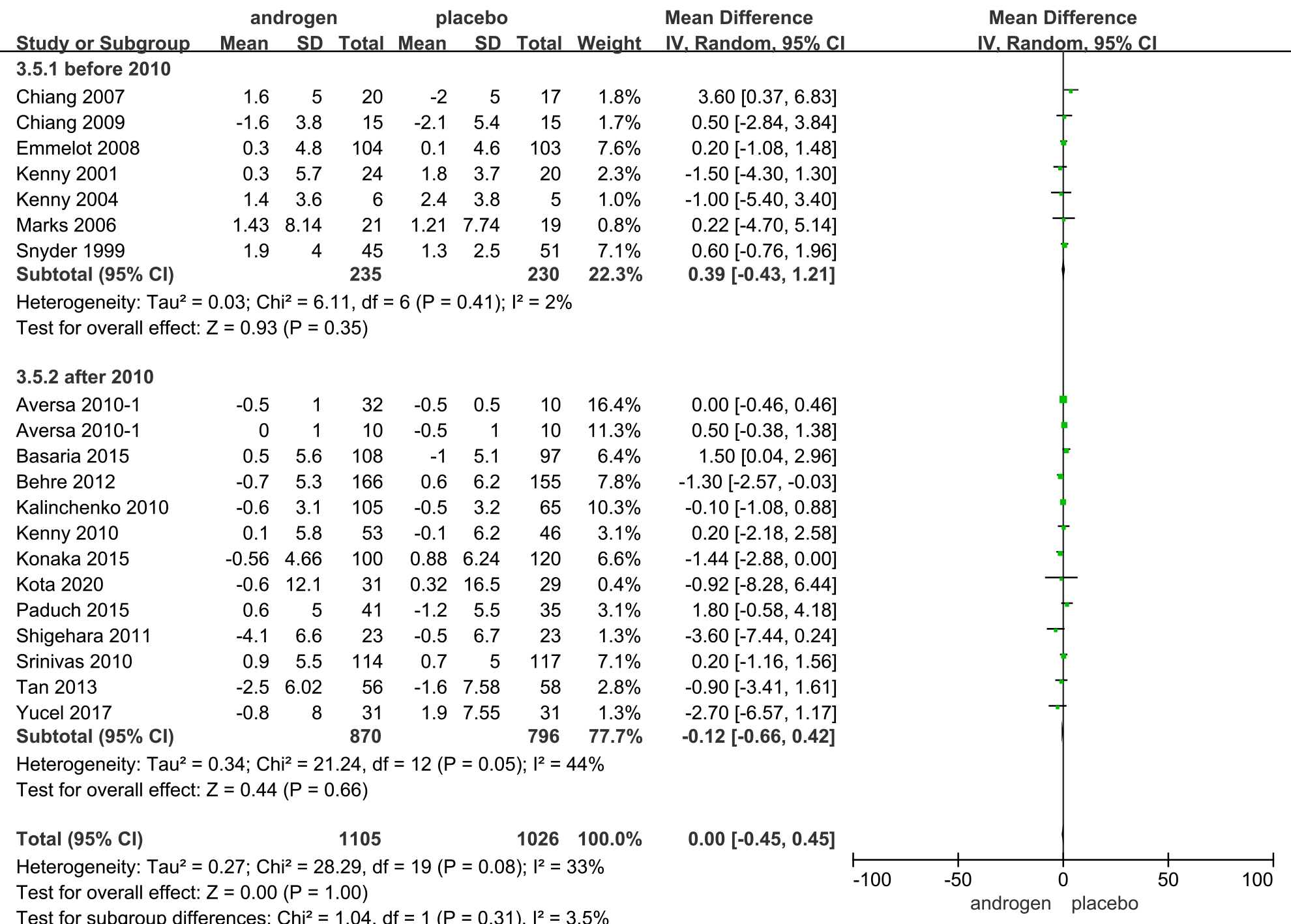

Supplement: Supplementary Figure 2 — Subgroup analysis of IPSS based on the year of publication. [file Image_2.tif]

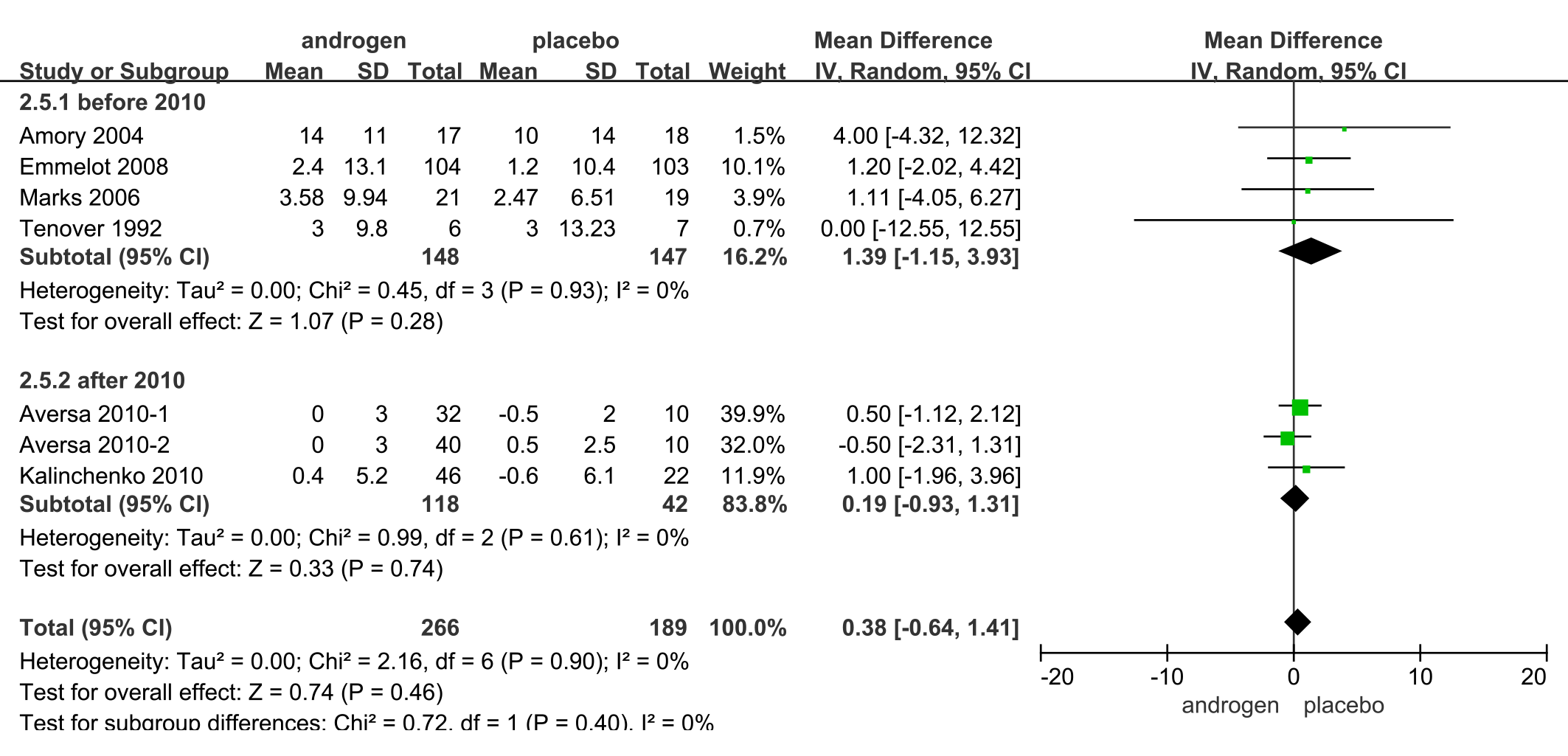

Supplement: Supplementary Figure 3 — Subgroup analysis of PV based on the year of publication. [file Image_3.tif]

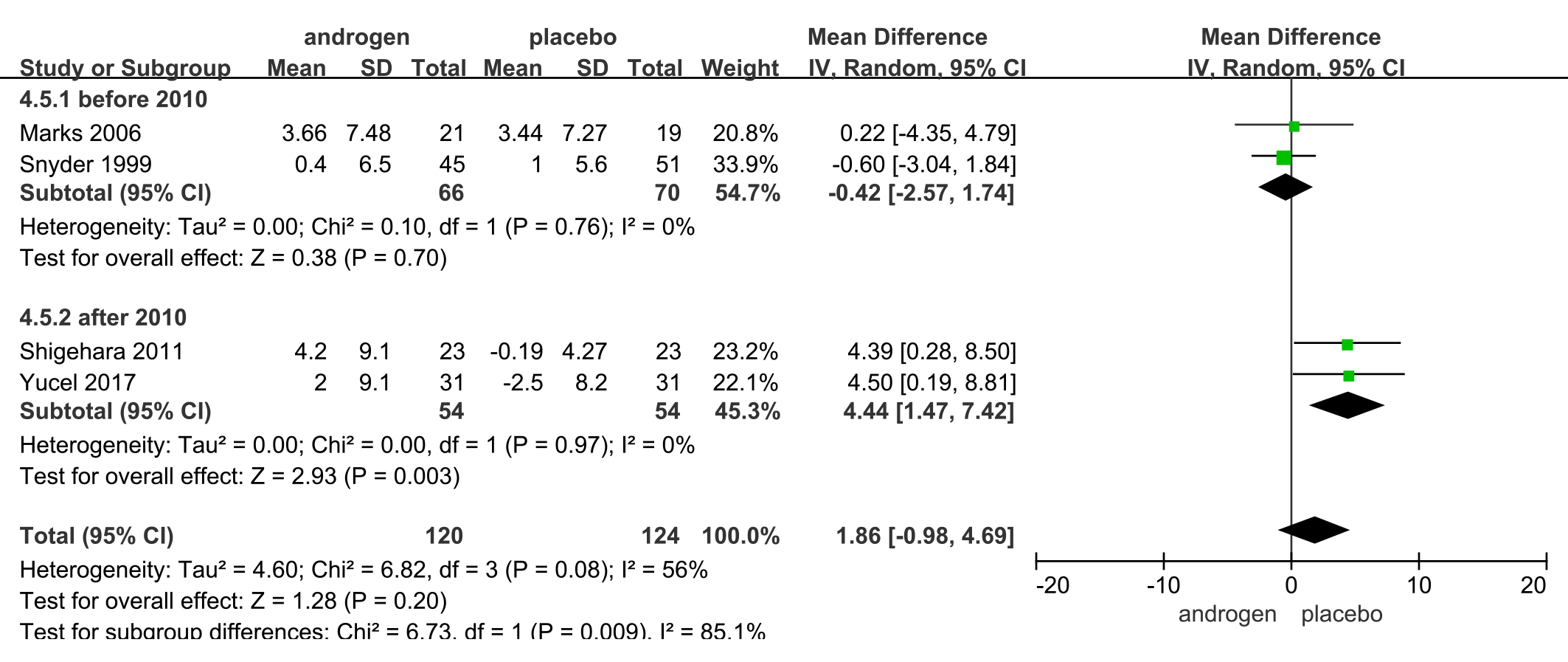

Supplement: Supplementary Figure 4 — Subgroup analysis of Qmax based on the year of publication. [file Image_4.tif]

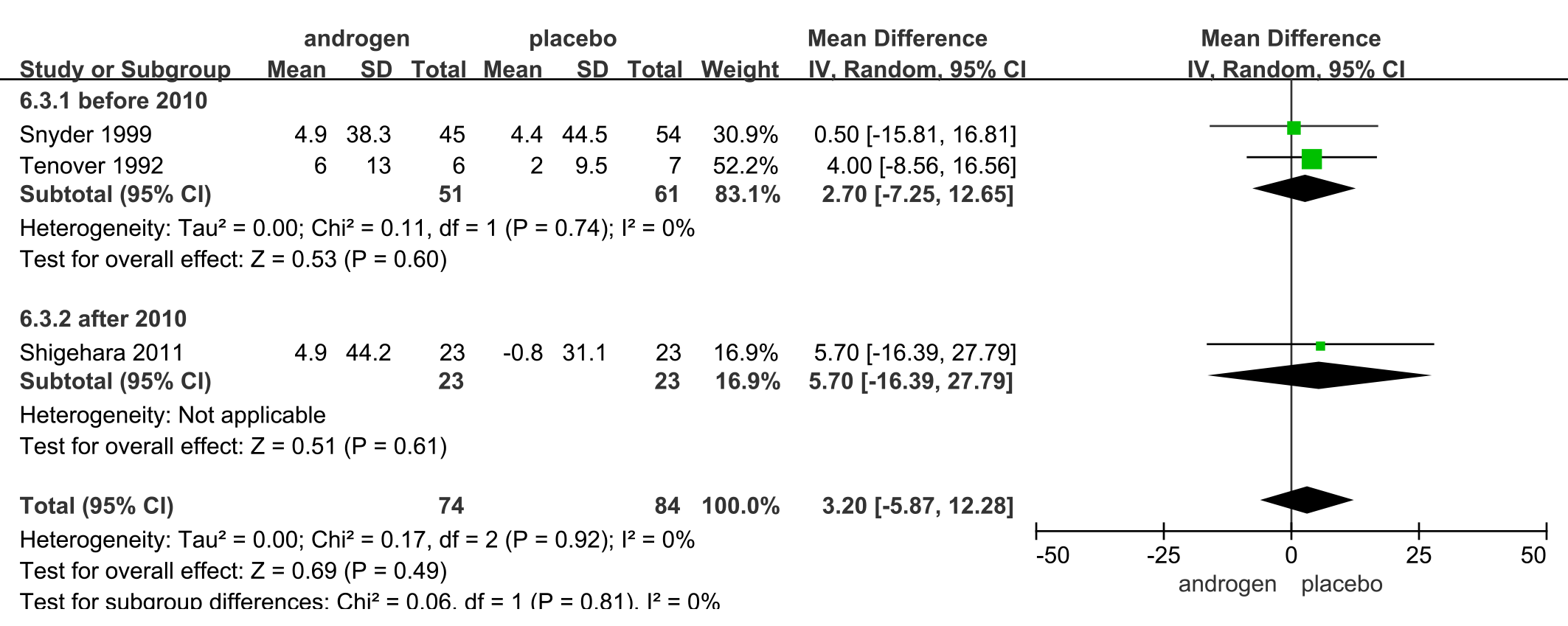

Supplement: Supplementary Figure 5 — Subgroup analysis of PVR based on the year of publication. [file Image_5.tif]

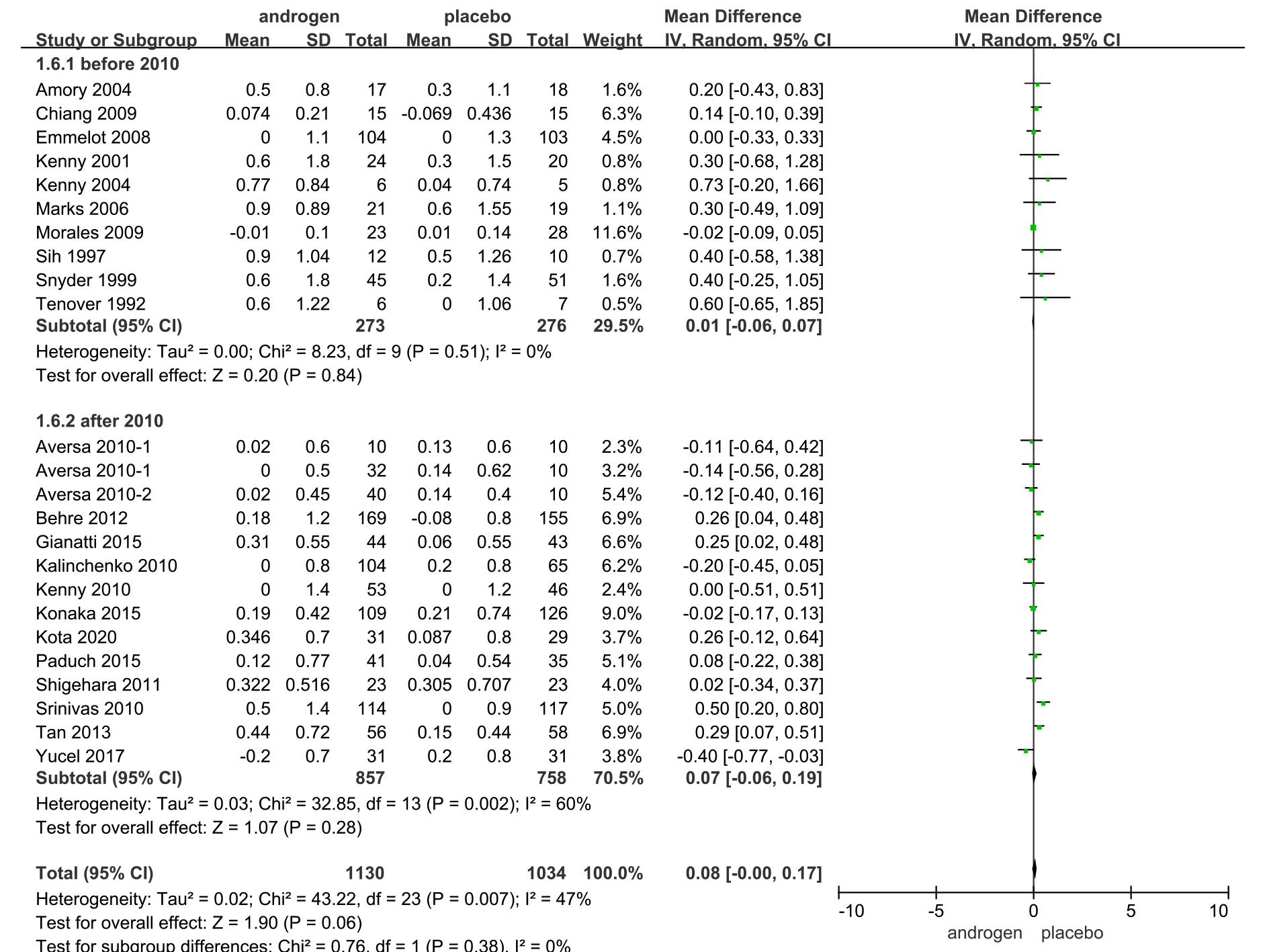

Supplement: Supplementary Figure 6 — Subgroup analysis of PSA based on the year of publication. [file Image_6.tif]
